# *Brucella melitensis* Biovar 1 and *Brucella abortus* S19 Vaccine Strain Infections in Milkers Working at Cattle Farms in the Khartoum Area, Sudan

**DOI:** 10.1371/journal.pone.0123374

**Published:** 2015-05-04

**Authors:** Amira E. F. Osman, Abdullahi N. Hassan, Ali E. Ali, Theresia H. Abdoel, Henk L. Smits

**Affiliations:** 1 University of Alzaiem Alazhari, Khartoum, Sudan; 2 KIT Biomedical Research, Royal Tropical Institute, Amsterdam, The Netherlands; Texas A&M Health Science Center, UNITED STATES

## Abstract

**Background:**

Human brucellosis is a preventable zoonoses that may become persistent, causing, if left untreated, severe localized disease. Occupational exposure to infected animals or animal products and consumption of fresh contaminated dairy are main risk factors.

**Methods:**

One hundred farmworkers employed at two cattle farms one in Khartoum North and one in Omdurman were screened for the presence of specific antibodies and seropositive workers were invited to donate a blood sample for blood culture. Molecular typing was used to characterize *Brucella* isolates.

**Results:**

Ten percent of farmworkers tested seropositive and while *Brucella melitensis* biovar 1 was isolated from the blood of three individuals, an isolate identical to the *B*. *abortus* S19 vaccine strain was isolated from a fourth person. All four bacteremic individuals were employed as milkers and did not have obvious disease.

**Conclusions:**

The isolation of the highly infectious pathogen *B*. *melitensis* from seropositive workers is consistent with the notion that the pathogen may persist in the blood without causing overt disease. While vaccination with strain S19 is essential for the control of bovine brucellosis the vaccine strain may be transmitted to the human population and protective measures remain important to prevent exposure also in view of the presence of *B*. *melitensis*. To create awareness for this potentially severe disease more information on the prevalence of the pathogen in different risk groups and in livestock in the Sudan is needed.

## Introduction

Brucellosis is an highly infectious and contagious zoonotic disease feared for its debilitating and incapacitating character [1]. Brucellosis is one of the most important zoonoses and is common in countries and communities where a large proportion of the inhabitants is involved in livestock farming activities, people live in close contact with their animals or consume raw milk and other dairy products prepared from fresh milk. Until recently the disease received very little attention as a cause of illness in African countries [2,3]. Brucellosis is caused by infection with slow-growing, small, Gram negative, cocco-bacilla bacteria of the genus *Brucella* [4]. Infection of livestock is associated with infertility, late term abortions, birth of weak calf, reduced milk production, and in males with orchitis and epididymitis [5,6]. Human brucellosis is a febrile disease capable of masquerading as a myriad of entities, both infectious and non-infectious [7]. The disease may affect any organ system and has a tendency towards chronicity and persistence. Brucellosis is difficult to diagnose requiring laboratory confirmation and treatment demands prolonged use of a combination of antibiotics with in some severe cases surgery [8].

On the basis of pathogenicity, host preference and phenotypic characteristics, eleven *Brucella* species are recognized of which four, *Brucella melitensis*, *B*. *abortus*, *B*. *suis and*, *more rarely*, *B*. *canis*, cause disease in human beings [9]. Each of these species have different livestock species as their preferred host which for *B*. *abortus* is cattle, for *B*. *melitensis* goat and sheep, for *B*. *suis* swine and *B*. *canis* infects dogs. *Brucella* species are further sub-divided in subtypes or biovars [10]. Although *B*. *melitensis* is more infectious and more often causes disease in human beings compared to *B*. *abortus* no difference in disease presentation and severity is observed when comparing patients infected with the two pathogens [7].

The first report on the presence of brucellosis in the Sudan dates back from 1908. Since then a series of studies have provided serological evidence for infection in cattle, goat, sheep and camels in different parts of Sudan [11,12]. Much fewer studies have addressed the presence of brucellosis in the human population of the Sudan. A low seroprevalence rate (1%) was reported for occupational contacts including butchers, slaughterhouse workers, milkers and cow attendants in Kassala State in Central Sudan [13]. In a more recent study a seroprevalence of 9% was reported for abattoir workers and the seroprevalence in camel nomads was as high as 60% [14]. The identification of risk groups and knowledge of the species that is causing disease in the human population is important for the development of a control policy, surveillance strategy and implementation of preventive measures [15]. Vaccination of livestock is the cornerstone for the control and prevention of brucellosis and effective and cheap life attenuated vaccines are available including S19 for cattle, and Rev-1 for goat and sheep [16]. In this study we investigate the presence of brucellosis among farmworkers employed at two cattle farms in Khartoum state and applied multiple-locus variable-number tandem repeat analysis (MLVA-16) for the identification of *Brucella* isolates cultured from the blood of seropositive individuals [17].

## Materials and Methods

### Ethics Statement

The study protocol was approved by the Ethics Committee of the Institutional Review Board the University of Alzaiem Alazhari. An oral consent procedure approved by the Ethics Committee because of the limited impact of the data and sample collection procedure and illiteracy of part of the participants. The purpose of the study was explained to each participant and oral consent was recorded on the patient data sheet in the presence of one witness. Record sheets were kept in a secured place with access by the principal investigators only. Individuals who tested positive were referred for further medical examination.

### Inclusion of Participants

Between April—May 2010 twenty farmworkers working at a cattle farm in Omdurman and eighty farmworkers employed at a cattle farm in Khartoum North Sharg Alneel in Khartoum state were asked to voluntarily donate a blood sample for investigation for brucellosis. A brief questionnaire was completed for all participants. Farmworkers were randomly selected and all 100 workers agreed to participate.

### Laboratory Testing

Serological testing in the Rose Bengal test and in the Serum Agglutination Test (SAT) with BA and BM antigen was performed according to routine diagnostic procedures [18,19]. The *Brucella* IgM/IgG lateral flow assay (LFA) which consists of two cassettes, one for the detection of specific IgM antibodies and the other for the detection of specific IgG antibodies was performed as described [20]. Briefly, 5 μL serum was placed into the sample port of the assay device followed by the addition of 130 μL running buffer supplied with the test. Test results are read after 10–15 min by visual inspection for a stained *Brucella* antigen test line in the viewing port of the assay device. LFAs were scored positive when staining at the test and control lines was observed and scored negative when the test line remained unstained.

For blood culture 7.5 ml freshly collected venipuncture blood was mixed with 1.5 ml sodium citrate and 20 ml distilled water, centrifuged at 2,000 x g for 30 min and the pellet was cultured on both tryptose agar and *Brucella* agar (Hi medium) with supplements in 10% CO_2_ at 37°C for up to 2 weeks before considering negative for *Brucella* [21]. Blood culture was performed for seropositive individuals.

### DNA Extraction and Multiple Locus Variable Number Tandem Repeat Analysis

Culture isolates were inactivated by suspending one loop of a solid bacterial culture in 200 μl DNA storage and extraction buffer consisting of 5.25 M GuSCN, 20 mM EDTA, 1.3% (wt/vol) Triton X-100 and 1 mg/ml alpha-casein in 50 mM Tris.HCl (pH = 6.4). DNA extraction was performed as described by Boom and coworkers [22]. PCR based on multiple locus variable number tandem repeat analysis (MLVA) genotyping of *Brucella* isolates was performed with MLVA-16 panel 1 (bruce06, -08, -11, -12, -42, -43, -45 and -55) primer sets for species identification and MLVA-16 panels 2A (bruce18, -19 and -21) and 2B (bruce04, -07, -09, -16 and -30) primer sets for further subspecies differentiation [17]. PCR products were separated by electrophoresis on 2% (panel 1) or 3% (panel 2) agarose gels stained with ethidium bromide and viewed by UV illumination. The length of the PCR product was deduced in dependence of the expected tandem repeat unit by comparison with a 100 bp or a 20 bp molecular marker ladder. For each run, DNA control from two reference strains was carried along. MLVA-16 patterns were compared with isolates in the public database *Brucella* 2010 (http://mlva.u-psud.fr; accessed May 2012) using cluster analysis performed by unweighted pair group method with arithmetic mean (UPGMA) algorithm [23]. The distance between two genotypes is defined as the minimum number of changes in the number of repeats of any locus that converts one genotype to the other.

## Results

Blood samples collected from one hundred laborers working at two farms in the Khartoum area were investigated. The sera from 10 (10%) individuals tested seropositive for brucellosis and *Brucella* was successfully isolated from the blood of four of them (Table 1). The four culture positive individuals were males between 26 and 38 years old employed as cattle milkers of which one also milked goat. The six culture negative seropositive individuals included four milkers, one veterinarian and one veterinarian technician. All participants denied feeling ill and to have physical complaints and none of them had been diagnosed with brucellosis before.

**Table 1:** Patient characteristics, laboratory test results and risk behavior of brucellosis positive individuals.

	Details of subject			Risk behaviour				Laboratory test result						
Patient	Location of farm	Age	Gender	Occupation	Contact animal	Consumption of raw milk	Consumption of raw meat	Rose Bengal	SAT (BA)	SAT (BM)	IgM LFA	IgG LFA	Culture	Type of isolate
1	Khartoum North	36	male	Milker	Cattle	No	No	+	1:160	1:160	1+	3+	+	*B*. *abortus* strain BCCNVI (S19)
2	Khartoum North	28	male	Milker	Cattle	No	No	+	1:640	1:1280	1+	1+	+	*B*. *melitensis* strain BCCN96-22
3	Omdurman	26	male	Milker	Cattle	Yes	No	-	1:640	1:640	1+	1+	+	*B*. *melitensisstrain* BCCN96-22
4	Omdurman	28	male	Milker	Cattle and goat	Yes	Yes	+	-	-	1+	1+	+	*B*. *melitensis* s*train* BCCN96-22
5	Khartoum North	49	male	Veterinary doctor	Cattle	No	No	-	1:20	1:160	1+	2+	-	*NA*
6	Khartoum North	33	male	Milker	Cattle	No	No	+	1:1280	1:320	4+	0	-	*NA*
7	Omdurman	38	male	Milker	Cattle	No	Yes	+	1:80	1:40	3+	0	-	*NA*
8	Omdurman	54	male	Veterinary technician	Cattle, sheep, goat and donkey	Yes	No	+	1:80	1:320	2+	0	-	*NA*
9	Khartoum North	40	male	Milker	Cattle	No	No	-	1:160	1:40	1+	0	-	*NA*
10	Khartoum North	31	male	Milker	Cattle	No	No	-	1:40	1:80	1+	0	-	*NA*

*NA*, not applicable

MLVA-16 genotyping of the Brucella isolates showed that one isolate (strain BruS3) was identical to the B. abortus S19 vaccine strain (strain BCCNV1) [17]. The three other isolates all were identified as *B*. *melitensis* biovar 1 with a MLVA-16 pattern identical to strain BCCN96_22 isolated from a sheep in Israel in 1996 [17]. This strain is closely related (distance = 1) to strains that were isolated from sheep in South Africa and Spain between 1986 and 2000 [17] and differs from the *B*. *melitensis* Rev1 vaccine strain by the number of repeats at panel 2B locus 16 (distance = 5) and from the 16M reference strain by the number of repeats at the panel 2A locus 18 and the panel 2B loci 07, 09 and 16 (distance = 11).

## Discussion

All ten seropositive farmworkers employed at two farms in Khartoum state identified in this study including the four milkers with a positive blood culture were at work and did not present with obvious disease at the time the sample was collected. While brucellosis is feared because of its tendency to become chronic and the risk of severe disabling disease, *Brucella* spp. are able to survive by replication within macrophages, has a multiple mechanisms to evade the immune system and may persist in their host causing no or mild disease only. The finding of infected individuals without clear disease is not unusual and asymptomatic culture positive individuals have been previously identified by screening household members of brucellosis patients. Spink and Anderson [24] described one case identified in 1950. Two asymptomatic household members of patients with a *B*. *melitensis* biovar 3 infection one of whom was a breast feeding mother of a child with brucellosis and from whom the pathogen was isolated from breast milk were reported in a study performed in Turkey [25]. Two other asymptomatic *B*. *melitensis* blood culture positive household members from patients with brucellosis were described in a study from Peru [26]. Evidence for the presence of the pathogen in the blood of occupational exposed asymptomatic individuals was obtained previously in studies applying the polymerase chain reaction for the detection of the DNA of *Brucella* [27,28]. Individuals with a latent subclinical infection are at risk of developing severe disease and should be treated. Therefore, the finding of culture positive farmworkers is worrisome given the limited awareness of clinicians in the Sudan to diagnose the disease and of the ignorance of the general population to protect from exposure. Brucellosis is rarely diagnosed in Sudan and must be confirmed by laboratory testing even symptomatic patients likely do not receive appropriate treatment. While the culture positive individuals did not report a history of brucellosis the possibility exists that brucellosis was misdiagnosed during a previous disease episode and evolved without severe sequellea or that an early disease episode was mild and medical care was not sought. Symptoms and signs of brucellosis are highly diverse and brucellosis is impossible to diagnose without laboratory conformation [1,7].

The detection of *Brucella* culture positive individuals by screening occupational exposed farmworkers indicates that brucellosis is a common but underdiagnosed infection in the Sudan. In this study we applied a very simple and rapid field test, *Brucella* IgM/IgG lateral flow point-of-care test, for the serodiagnosis of brucellosis [1]. The test is applied on a drop of blood collected by finger prick, does not requires specific equipment or training to perform and provides a very quick result [19]. The test could be very useful for the confirmation of brucellosis in hospitals that do not have laboratory facilities and to provide medical services in the field for patients who do not have easy access to medical services [26,29].

Close contact with infected livestock is a major risk factor for attracting brucellosis and milk collected from infected animals may contain high concentrations of the pathogen. Two of the culture positive individuals reported consumption of raw milk and one ate raw meat. Thus the farmworkers could have been exposed by different routes, including close contact with infected animals, exposure to contaminated milk and ingestion of raw foodstuff and the provision of information to farmworkers in Sudan on the use of protective measures will be important.

Cattle at the farms included in this study had been vaccinated with the S19 vaccine by teams of the by the Ministry of Animal Resources. The S19 vaccine is a live attenuated vaccine that is contagious for human and cases of clinical disease has been observed upon accidental exposure to the vaccine in veterinarians and vaccine plant workers [30–32]. In a small proportion of vaccinated animals that aborted the vaccine strain can be isolated from abortion material [33]. S19 also may be isolated from the milk of vaccinated cows. Therefore, farmers and other workers who are involved in vaccination or handle vaccinated cattle and consumers who have consumed contaminated milk from vaccinated cows that has not been pasteurized have a slight risk of infection and attracting the disease. It is not known whether cattle was positive for *Brucella* at the time of vaccination.

The finding of *B*. *melitensis* in three farmworkers working at cattle farms is noteworthy. One of these farmworkers also milked goat which could have been the source of infection for that person. While the source of infection has not been investigated infection of cows with *B*. *melitensis* has been reported [34–37]. Transmission to cattle may occur when infected goats are kept together with cattle and mixed herding is an important risk factor in the transmission of *B*. *melitensis* from goat to cattle. If indeed *B*. *melitensis* is present in cattle in Sudan this will complicate the control of bovine brucellosis and may require an approach that also considers goats and sheep. The S19 vaccine does not protect cattle from infection with *B*. *melitensis* and the *B*. *melitensis* Rev-1 vaccine for the vaccination of goat and sheep has not been validated for use in cattle [37].

*B*. *melitensis* biovar 1 was not identified in Sudan before. Studies employing culture have demonstrated the presence in Sudan of *B*. *melitensis* biovar 3 in sheep and goat, *B*. *abortus* biovar 6 in cattle and *B*. *abortus* biovar 3 and biovar 6 in camel [38,39].

In conclusion, isolation of *Brucella* from 4% of milkers presents an unacceptable health risks and efforts should be undertaken to provide health education and to implement preventive measures. The isolation of *B*. *melitensis* from cattle milkers suggests that the species is transmitted to cattle. The isolation of the *B*. *abortus* S19 vaccine strain from the blood of one farm employee raises concern about the safe use of the vaccine. Further studies are warranted to investigate the presence of *Brucella* in different livestock species in the Khartoum area and to determine the prevalence of brucellosis in different risk groups and hospitalize patients. Information on the prevalence of brucellosis and importance of the disease as a public health problem in Sudan is essential to create awareness and to improve early case detection. The use of a rapid and simple point-of-care test such as the *Brucella* IgM/IgG lateral flow assay could be very useful to provide medical services to hard-to-reach populations and to improve early case detection [25,29].
